# Effect of Diameter and Type of Suture on Knot and Loop Security

**DOI:** 10.3390/jcm12196418

**Published:** 2023-10-09

**Authors:** Armando Romeo, Christiane Fujimoto, Isabella Cipullo, Mauricio Giarola, Chiara Benedetto, William Kondo, Bedayah Amro, Anastasia Ussia, Arnaud Wattiez, Philippe R. Koninckx

**Affiliations:** 1 Research Educational Center, University of Turin, 10124 Turin, Italy; armandoromeo56@gmail.com; 2 Department of OBGYN, Faculty of Medicine, University of Santa Casa di Sao Paulo, Sao Paulo 05508-220, Brazil; chrisfuji@terra.com.br; 3 Department Obstetrics and Gynecology, Ospedale Regina Montis Regalis di Mondovìn, 12084 Turin, Italy; cipulloisabella@gmail.com (I.C.); chiara.benedetto@unito.it (C.B.); 4 Department of OBGYN, Faculty of Medicine, S.Anna Hospital Torino, 10126 Turin, Italy; mauriziogiarola@libero.it; 5 Centro Avançado de Cirurgia Ginecológica, Department of OBGYN, Faculty of Medicine, Curitiba 81020-430, Brazil; williamkondo@gmail.com; 6 Department of OBGYN, Faculty of Medicine, Latifa Hospital, Dubai 9115, United Arab Emirates; bedaya1984@yahoo.com (B.A.); arnaud.wattiez@wanadoo.fr (A.W.); 7 Department of OBGYN, Faculty of Medicine, Ucatholic University and Gruppo Italo Belga, Villa Del Rosario, 00168 Rome, Italy; anastasia.ussia@gmail.com; 8 Department of OBGYN, Faculty of Medicine, University of Strasbourg, 67081 Strasbourg, France; 9 Department of OBGYN, Faculty of Medicine, Katholieke University Leuven, 3000 Leuven, Belgium; 10 Department of OBGYN, Faculty of Medicine, University of Oxford, Oxford OX1 2JD, UK; 11 Department of OBGYN, Faculty of Medicine, University Cattolica, del Sacro Cuore, 00168 Rome, Italy

**Keywords:** knot sequences, half-knots, half-hitches, knot rotation, knot security, loop security, laparoscopic surgery

## Abstract

The loop and knot securities of two polyfilament and two monofilament sutures of four diameters (3.0, 2.0, 0, 1) were evaluated with a tensiometer for four four-throw knots, known to be secure with a 2.0 polyfilament suture. Loop security of Monocryl 1 is low, being 14.7 ± 3.0 Newton (N) for a three-throw half-knot (H3) and 15.4 ± 2.4 N and 28.3 ± 10 N for two (SSs) and four (SSsSsSs) symmetrical sliding half-hitches. This is lower than 18, 24, and 46 N for similar knots with Vicryl. Polyfilament sutures have excellent knot security for all four diameters. Occasionally, some slide open with slightly lower knot security, especially for larger diameters, although this is not clinically problematic. Knot security of monofilament sutures was unpredictable for all four knots, especially for larger diameters, resulting in many clinically insecure knots. A secure monofilament knot requires a six-throw knot with two symmetrical sliding half-hitches or two symmetrical half-knots secured with four asymmetric blocking half-hitches. In conclusion, with polyfilament sutures, four- or five-throw half-knot or half-hitch sequences result in secure knots. For monofilament sutures, loop and knot security is much less, half-knot combinations should be avoided, and secure knots require six-throw knots with four asymmetric blocking half-hitches.

## 1. Introduction

Suturing and knot tying are basic skills in surgery. Knot tying is defined by the knot and loop security, defined as the forces at which the suture breaks or slides open and measured with a tensiometer [1,2,3,4,5]. Knot security is the resistance to opening the completed knot and thus reflects the forces needed to keep tissues together after surgery. Loop security is the resistance to sliding open the first throw of half-knots or one or more sliding half-hitches. Loop security is the force that keeps the edges of the tissue approximated or a vessel leak-proof until the knot is completed [4,6]. Loop security is more important in laparoscopic surgery than in open surgery because two-hand knot tying, permitting to keep traction on both suture ends, is not possible.

**Figure 1:**
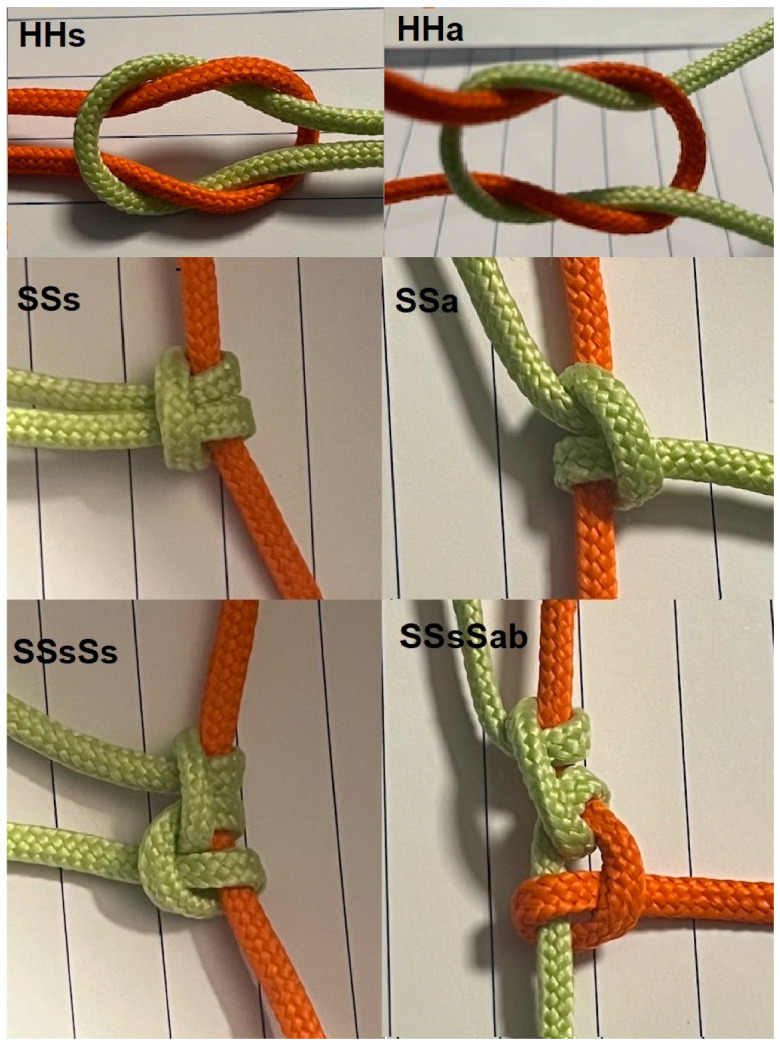
Symmetric (HHs) and asymmetric (HHa) half-knots are easily changed into half-hitches (SSs and SSa, respectively). Symmetric half-hitches (SSs or SSsSs) remain in one plane, with both ends on one side squeezing the passive end symmetrically and firmly. When the active and passive ends are changed for a blocking half-hitch, the asymmetric half-hitch becomes symmetric on the new passive end (SSsSab).

Loop and knot security are known to vary with the total number of throws and the number and rotation of half-knot or half-hitch sequences [7]. However, only recently were the mechanisms understood for 2.0 polyfilament sutures as reviewed in [8]. The observations can be summarised as follows. Surgical knots are sequences of half-hitches (S) or half-knots (H) (Table 1). Half-hitches result from pulling one end of the suture, called the passive end. Half-knots (H) require symmetrical pulling of both ends. Half-hitches usually consist of one throw and half-knots of one to three throws. Loop security of half-knots increases with the number of throws [8]. Loop security of sliding half-hitches increases with the number of half-hitches and with alternate rotation. Rotation is alternate when the rotation of the same active end is the opposite compared to the previous one. Half-hitch sequences with opposite rotation remain in one plane, with both ends on one side squeezing the passive end symmetrically and firmly. Knot security of half-knot and half-hitch sequences also varies with rotation [4,8,9,10]. Half-knot sequences with similar rotation result in an insecure granny knot, whereas opposite rotation results in a flat square knot, visually recognised as symmetrical in one plane [8]. Changing the active and passive ends, as conducted in bimanual suturing or when making blocking half-hitches, has a similar effect as changing rotation. Therefore, similar rotation improves knot security of blocking half-hitches or knots made with bimanual suturing [11] (Figure 1).

Besides rotation, knot security increases with the number of throws. With 2.0 polyfilament sutures, secure knots require four or five throws, i.e., a two- or three-throw half-knot, followed by a two-throw half-knot or a surgical knot, which is a two-throw half-knot followed by two symmetrical one-throw half-knots. Secure half-hitch sequences need two symmetrical half-hitches followed by two asymmetrical blocking half-hitches. For knots such as H2H2 and H3H2 sequences, rotation only marginally affects security, with asymmetrical sequences even being slightly better [8]. A different class of knots are cinch knots [12], which are complex sliding knots that can be blocked when in place by reorganising the knot structure by pulling the active end, such as the Röder knot [13].

Another clinically important aspect of knot security is the occasional reorganisation of the knot structure when forces are applied to secure the knot. Although this reorganisation can result in very low knot security, reorganisation has been poorly investigated because at least 40 knots are needed to detect 5% insecure knots. Clinically, the most important reorganisation results from the poor loop security of a one- or two-throw half-knot, which easily transforms into a one- or two-throw half-hitch. Because of the poor loop security, these half-knots are easily destabilised by tissue forces, by little involuntary traction on one of the ends of the first loop when making the second half-knot, or by not tying the second half-knot perfectly symmetrical. This risk increases when the surgeon is less experienced, the suture ends are short, or knot tying is difficult because it is deeper or less accessible. Because the loop security of a three-throw half-knot (H3) is better, the risk is lower. The clinical consequence of this involuntary 3D reorganisation is that the knot security of half-knot sequences can be variable with occasionally dangerous knots with a knot security of less than 5 or 10 N [8].

The forces of loop and knot security needed for different tissues in gynaecological and abdominal surgery have been poorly documented. Still, common sense suggests that during coughing, forces on the abdominal fascia or on the promontory after promontofixation must be higher than those needed for bowel or vaginal cuff suturing. The knot and loop securities of 2.0 polyfilament sutures are well documented. However, whether the results can be extrapolated to sutures with a smaller or larger diameter and to monofilament sutures with different sliding and friction characteristics in the tissue and the knot is unknown. Therefore, we investigated the loop and knot security of four excellent knot combinations for polyfilament and monofilament sutures of different diameters.

## 2. Materials and Methods

### 2.1. Terminology of Knots

Knot classification, tying, and testing were described in detail previously [8]. Knots are defined by their type, number of throws, and rotation (Table 1). An ‘**H**’ is used for half-knots and an ‘**S**’ for half-hitches, followed by the number of throws and the rotation of the active end in comparison with the previous one (‘**s**’ for symmetric knot resulting from alternate rotation and ‘**a**’ for asymmetric after similar rotation). In addition, for half-hitches, a sliding sequence is indicated with ‘**s**’ and blocking with ‘**b**’. This terminology can be simplified by omitting the number of throws if 1 and the **s** of sliding to avoid confusion with symmetric. It is important for surgeons to realise that changing the active and passive ends, as occurs with bimanual suturing or when transforming a sliding in a blocking half-hitch, has the same effect as changing rotation. Thus, SSsSsbSab means a second symmetrical and sliding half-hitch (made by alternate rotation). The third half-hitch is also made by alternate rotation (and is therefore called symmetric), resulting in an asymmetric blocking half-hitch of lower quality after changing the active and passive ends. The third half-hitch was made by similar rotation (and is therefore called asymmetric), resulting in a symmetric blocking and stronger half-hitch after changing the active and passive ends.

This terminology emphasises rotation because the surgeon performs this when tying knots. He only has to remember that alternate rotation of the active end is the basic rule because it results in symmetric knot sequences, which are superior to the asymmetric knot sequences made by a similar rotation. The only exception is blocking half-hitches: he has to start with a similar rotation (asymmetric) because after changing the active and passive ends (with a similar effect as changing rotation), the asymmetric (sliding) half-hitch becomes a symmetric blocking half-hitch on the new passive end. This terminology is less confusing than the previous one (Table 1), describing the final knot sequence as symmetric with ‘=’, asymmetric with ‘x’, and blocking with ‘//’ without mentioning rotation.

### 2.2. Aim of the Study, Knot Combinations, and Power Estimations

This study investigated the effect of suture diameter (suture sizes) and type of suture (polyfilament or monofilament) on loop and knot security. Because loop security was documented only with polyglactin 2.0 (polyfilament), we measured loop security with a monofilament (Monocryl) for half-knots (H2, H3), half-hitches (2, 4, or 6 symmetrical half-hitches), 2 symmetrical half-knots (H1H1s), and a 2-throw half-hitch (S2). For knot security, we investigated 4 diameters (3.0, 2.0, 0, and 1) of 2 polyfilament sutures (Vicryl, polyglactin and Mersilene, polyethylene terephthalate) and 2 monofilament sutures (PDS, polydioxane and Monocryl, lubricant-coated poliglecaprone) for 4-throw knot sequences, considered secure knots with Vicryl, such as SSsSabSab, H2H2a, H2H2s, and H2H1sH1s. Because of the high incidence of insecure 4-throw knots with larger monofilament sutures, 5-knot sequences were investigated, such as 3 or 2 sliding with 2 or 3 blocking half-hitches (SSsSsSabSab and SSsSabSabSab), the sliding H1H1s with 3 blocking half-hitches (H1H1sSabSabSab), and the S2 with 3 blocking half-hitches (S2SabSabSab).

All knots were made by the same experienced person (AR) with close supervision of eventual mistakes by IC, and knots were block-randomised for each experiment. Thus, for four groups, 1 knot of each group was made before starting the second series. A factorial design was used with 10 knots for each type of suture.

Institutional review board (IRB) approval was not needed for experiments in vitro not involving humans or animals, as confirmed in writing by the IRB of Leuven University.

### 2.3. Knot Tying and Testing

Using Romeo’s gladiator knot-tying technique, standardised laparoscopic knots were made as described [8]. Sutures of 18 cm were tied around a 15 mm plastic tube using the different knot combinations to be evaluated. After knot tying, the suture threads were cut at exactly 10 mm to permit the detection of sliding. These loops were subsequently mounted on the hooks of a digital dynamometer (Sauter FH 500 capacity 500 NW) and tested at 200 mm/min. With increasing forces, the knot combination slipped open or blocked, causing the suture to break. The two endpoints thus are breaking or sliding open and the force (N) at which the knot slips open or breaks. Testing was conducted at the Research Educational Centre of Turin University.

### 2.4. Statistics

Statistical evaluation was conducted with SAS 9.4 TS1M3 [14] and means and SDs are given unless indicated otherwise. A factorial design with 10 knots in each cell, 4 types of sutures, 4 diameters, and 4 knots has a statistical power of 540 knots, additionally permitting to evaluate interaction [15]. Data were analysed by 2-way analysis of variance for non-Gaussian distributions (proc GLM).

Differences between 2 groups were evaluated with the Wilcoxon signed rank test.

## 3. Results

**Figure 2:**
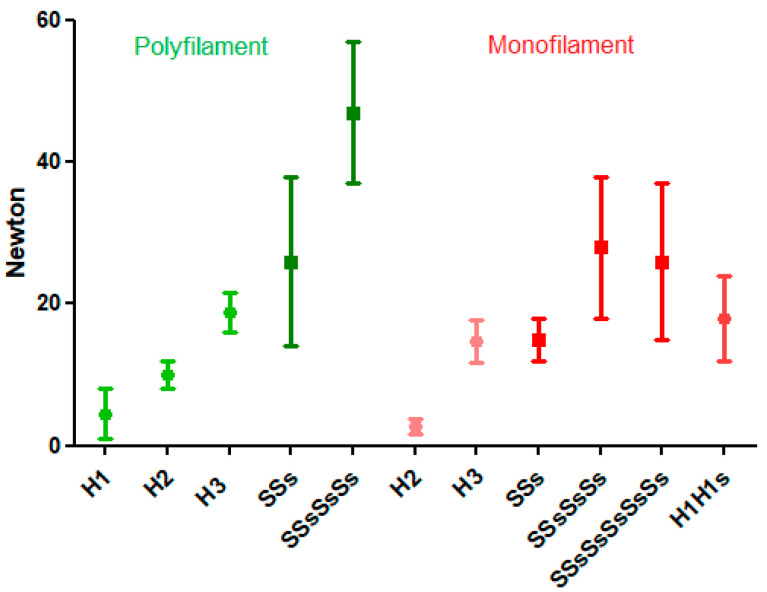
Loop security of polyfilament (Vicryl 2.0) and monofilament (Monocryl 1) sutures. Mean and SDs are indicated.

Loop security of Monocryl 1 is low, with large standard deviations indicating variability. Loop security is 2.7 ± 1.0 N for H2, 14.7 ± 3.0 N for H3, 15.4 ± 2.4 N for SSs, 28.3 ± 10 N for SSsSsSs, 26.5 ± 11.5 N for SSsSsSsSsSs, 9.9 ± 5.1 N for S2S1, and 17.7 ± 6.2 N for H1H1s (Figure 2).

**Figure 3:**
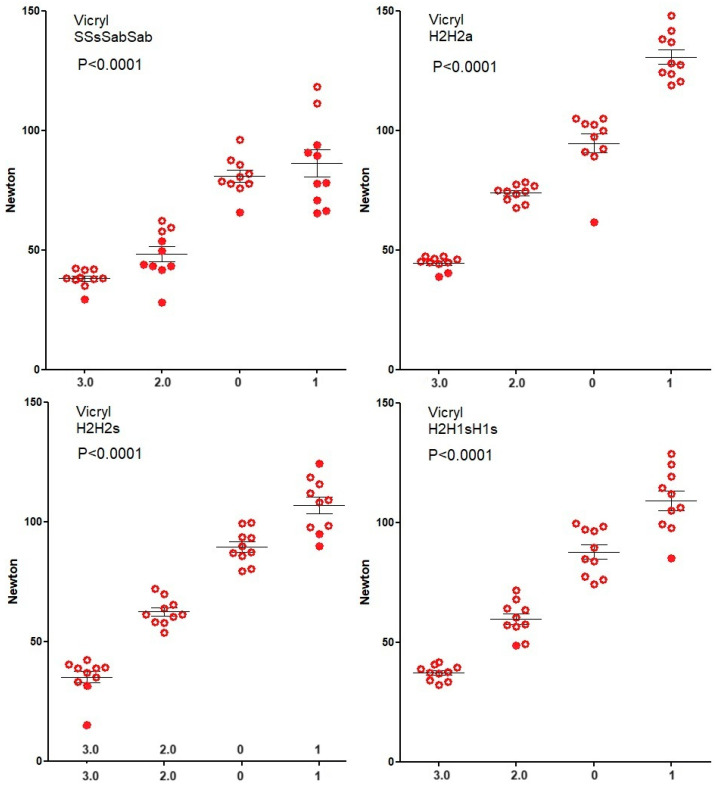
Knot security of Vicryl. Open circles indicate knots that break and closed circles indicate knots that slide open. Mean, SEM, and *p*-values for the correlation of knot security with suture diameter are indicated.

**Figure 4:**
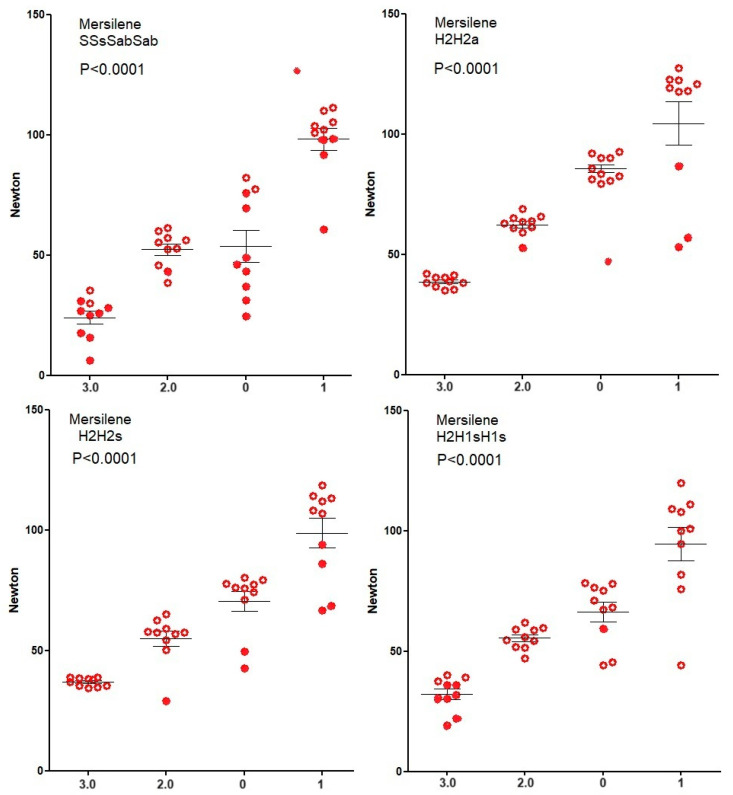
Knot security of Mersilene. Open circles indicate knots that break and closed circles indicate knots that slide open. Mean, SEM, and *p*-values for the correlation of knot security with suture diameter are indicated.

**Figure 5:**
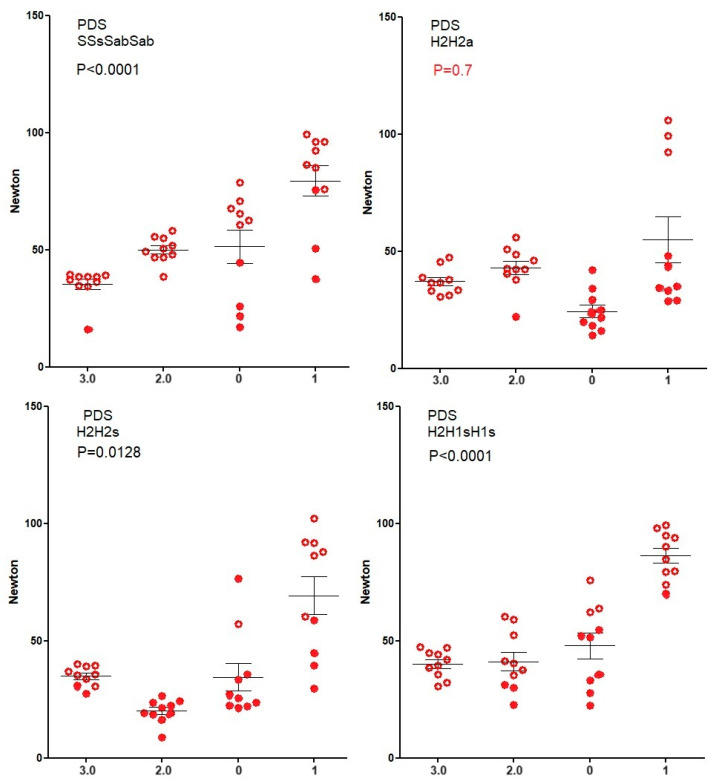
Knot security of PDS. Open circles indicate knots that break and closed circles indicate knots that slide to open. Mean, SEM, and *p*-values for the correlation of knot security with suture diameter are indicated.

**Figure 6:**
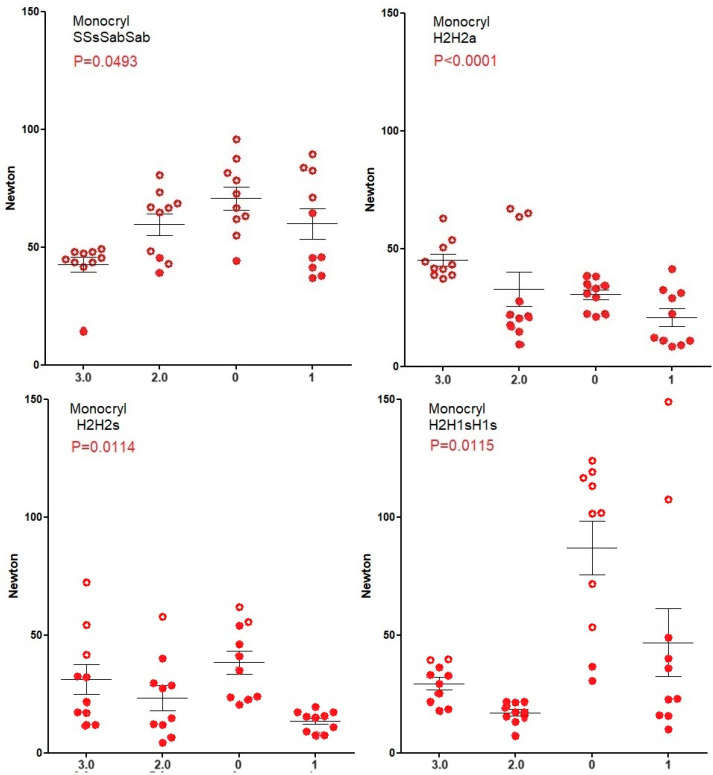
Knot security of Monocryl. Open circles indicate knots that break and closed circles indicate knots that slide open. Mean, SEM, and *p*-values for the correlation of knot security with suture diameter are indicated.

Knot security of Vicryl (Figure 3) and Mersilene (Figure 4) increase (Spearman) with suture diameter for all four knots (all *p* < 0.0001) because breaking forces of sutures increase. However, for PDS (Figure 5), knot security only increases with suture diameter for SSsSabSab (*p* < 0.0001), H2H2s (*p* = 0.0128), and H2H1sH1s (*p* < 0.0001) but not for H2H2a (*p* = 0.7). For Monocryl (Figure 6), knot security slightly increases only for SSsSabSab (*p* = 0.0493) and H2H1sH1S (*p* = 0.0115) and decreases for H2H2 (*p* < 0.0001) and H2H2s (*p* = 0.0114). This is caused by the unpredictable number of knots sliding open, being 22% for polyfilament and 51% for monofilament sutures (<0.0001) and increasing when suture diameters are larger (all *p* = 0.0171). However, this increase with diameter is not consistent neither for the type of sutures (Vicryl *p* < 0.0001, PDS *p* = 0.0003, Monocryl *p* < 0.0001, mercilene *p* = 0.67) nor for the knot combination (SSsSabSab *p* = 0.4692, H2H2a *p* = 0.0001, H2H2s *p* = 0.1184, H2H1sH1s *p* = 0.1340). Also, the excellent H3H2a knot with Vicryl results in many insecure knots with Monocryl 1).

All two- or three-way analyses of variance simultaneously comparing knot types, diameter, and mono- or polyfilament sutures (n = 320) confirmed that knot security increases with the diameter (*p* < 0.0001) and when using polyfilament (*p* < 0.0001) for all knot types. The percentage of knots sliding open also increases with the diameter (*p* = 0.0124) and when using monofilament sutures (*p* < 0.0001). With polyfilament sutures, the security of the four types of knots was clinically comparable, notwithstanding minor differences (all *p* < 0.0001 except between H2H2s and H2H1sH1s). With monofilament sutures, SSsSabSab was superior to the half-knot combinations for knot security (*p* < 0.0001 but *p* = 0.076 versus H2H1sH1s) with fewer knots sliding open (*p* < 0.0001 and *p* = 0.0004 versus H2H1sH1s).

**Figure 7:**
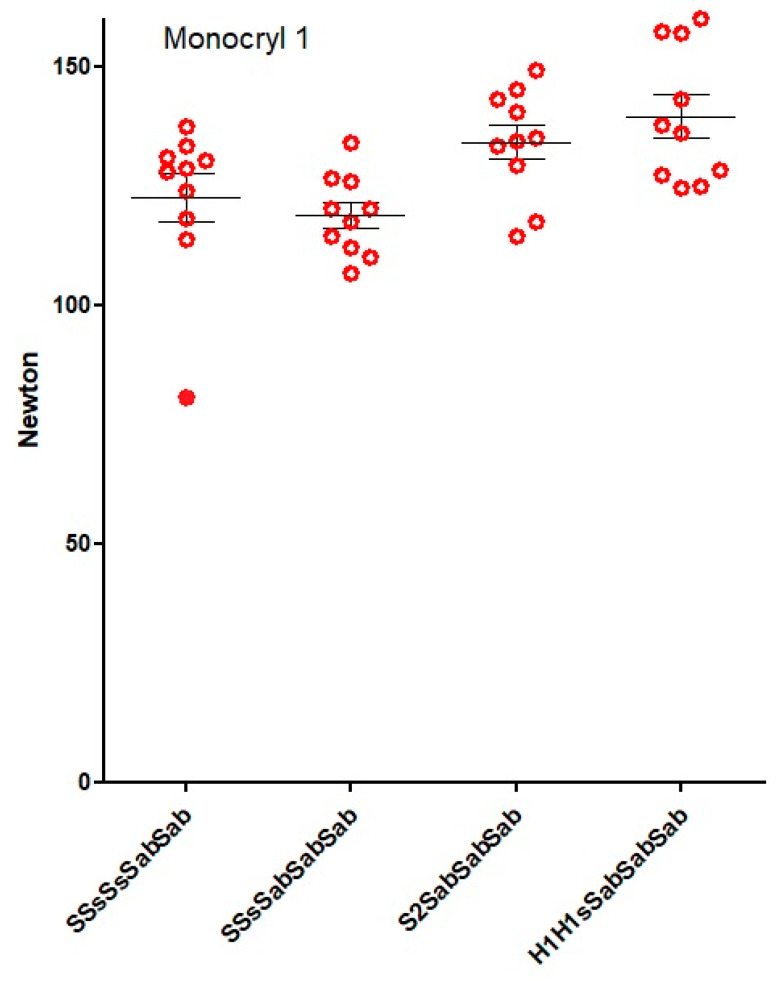
Knot security of Monocryl 1 for 5-throw knots composed of 3 sliding + 2 blocking, 2 sliding + 3 blocking, 2-throw + 3 blocking half-hitches, and an asymmetrical square knot + 3 blocking half-hitches.

To investigate which knot combinations resulted in reliable monofilament sutures, five-throw combinations were investigated (Figure 7). Surprisingly, all five-throw combinations with three blocking half-hitches were secure irrespective of the base being SSs, H1H1s, or S2.

## 4. Discussion

Surprisingly, after so many years of surgery and more than 30 years after the introduction of the tensiometer, the definitions and our understanding of loop and knot security are still limited. Traditionally, with open surgery, bimanual knot tying was used to permit constant traction on both suture ends, thus preventing loop instability, and the reliability of the surgical knot (H2H1sH1s) was not questioned. In laparoscopic surgery, loop stability became an issue because bimanual knot tying was no longer possible. Especially the higher forces in orthopaedic endoscopic surgery needed a high loop security for tight knots.

For loop security, the choice of knot sequences varies with the estimated forces needed to keep edges approximated or a vessel leak-proof. For loop security, a polyfilament suture is preferable because the loop security of monofilament sutures is low. Although the loop security of two symmetrical half-hitches is not superior to a three-throw half-knot, half-hitches are preferred because they add versatility. If needed, a third or a fourth half-hitch can be added, and the last can eventually be changed into a blocking half-hitch.

Understanding knot security and the sequences of half-knots or half-hitches to be used has two important clinical consequences. The rare, but important, occasional wound dehiscences will decrease or disappear because they are probably caused by insecure knots caused by less adequate sequences or reorganisation. Also, postoperative adhesion formation will decrease because knowledge of knot security will permit cutting the tails of the knots shorter and using thinner sutures. A lower knot volume and shorter tails will reduce postoperative adhesions [16]. Without understanding knot security, suture ends used to be cut at 1 cm from the knot, reflecting the clinical experience of knots occasionally sliding open. Also, the diameter of the sutures used seems often exaggerated, considering the tensile strengths of sutures and the forces needed to keep the tissues approximated.

These considerations are even more important for monofilament sutures, which are developed to cause less of a tissue reaction, decrease the infection risk, and combine easy tissue sliding with high tensile strength. However, their loop or knot security characteristics were poorly investigated, assuming they would be similar to polyfilament sutures. Our data demonstrate that this is not the case and that monofilament sutures require specific knowledge of loop and knot security to achieve reliable suturing. The low loop security of monofilament sutures, especially when large diameters are used, suggests that symmetric half-hitch sequences are to be preferred. The knot security of four-throw knots, considered excellent with polyfilament sutures, is poor. Monofilament sutures need at least five-throw knots with three asymmetric blocking half-hitches. This is consistent with the report that 10% of four-throw half-knots (H1H1sH1sH1s) with PDS opened and that even six-throw half-knots (H2H1sH1sH1s H3H2sH1sH1s) were insecure [17].

Clinically, the choice of the suture diameter, type of suture, and knot sequences should be based on the forces estimated to be needed for loop security and knot security. Knot security should be reliable and predictable, without exceptions. Therefore, knot stability should not be estimated by the mean resistance to opening or by the percentages of sutures breaking because these endpoints do not indicate the number of insecure or dangerous knots. A reliable knot security determines the choice of the knot sequences to be used in surgery. With polyfilament sutures, all four-throw half-knots, such as the surgical knot and the H2H2, are reliable, but half-hitch sequences require an extra throw for security. Monofilament sutures require a five- or six-throw knot with at least three or better four blocking half-hitches following a two-throw basis. With monofilament sutures, half-knots should be avoided.

Fortunately, the clinical choice of knot sequences does not change with the suture diameter. Although 22% of knots slide open with slightly lower knot security, with the risk increasing with suture diameter, this is clinically less important because it is rarely less than 50 N. This unpredictable behaviour is more frequent with monofilament sutures, and 51% of knots slide open, especially for larger suture diameters.

## 5. Conclusions

These results confirm [13] the superiority of half-hitches in gynaecological surgery. Half-hitch sequences are preferred when a high loop security is needed because of the traction of the tissues to be approximated. They not only have higher loop security, but they also add flexibility. If the loop security of two sliding half-hitches is insufficient, a third symmetrical sliding half-hitch, and if still insufficient, a fourth asymmetrical sliding half-hitch can be added. The latter can, if necessary, be transformed into a blocking half-hitch by quickly changing the active and passive ends.

Secure knots with polyfilament sutures require at least four throws, and H2H1sH1s, H2H2a, H2H2s, and SSsSabSab have comparable knot security. Monofilament sutures require at least a five-throw knot with three asymmetrical blocking half-hitches for a secure knot. Half-hitches, moreover, have a lower risk of being destabilised during knot tying. Therefore, they are the preferred knots in deep gynaecological suturing with the simple rule of alternative rotation for sliding and similar rotation for blocking sequences. These are minimal requirements. It remains a clinical judgment to add an extra half-hitch or half-knot, which is an additional argument for using half-hitches instead of half-knots.

## Figures and Tables

**Table 1:** Terminology of knot sequences. Following the H for half-knots or the S for half-hitches, the number of throws is indicated. Next, the result of the rotation in comparison with the previous knot is given. A similar rotation results in an asymmetric knot (a) and an opposite rotation in a symmetric knot (s), as shown in Figure 1. H1H1s is thus a flat square knot and H1H1a a granny knot. With polyfilament sutures, secure half-knot sequences are the surgical knot (H2H1sH1s) and 2 or 3-throw half-knots followed by a 2-throw half-knot, irrespective of rotation. The number of throws is omitted for half-hitches because it is always 1, but the sequence blocking (b) after changing the active and passive ends is indicated. A sliding (s) sequence is not indicated to avoid confusion with the s of symmetric. However, changing the active and passive ends has the same effect as changing rotation, and an asymmetric half-hitch becomes symmetric on the new passive end. Knot sequences used to be indicated with = for symmetric (also called identical), x for asymmetric, and // for blocking. We prefer a more intuitive indication emphasising rotation because this is what the surgeon performs when making knots, and sliding is not indicated to avoid confusion with the s indicating symmetric. A 2-throw half-hitch results from the transformation of a 2-throw half-knot.

Knots	Throws	Knot Sequences	Rotation	Old Terminology	New Terminology
**Half-knot**	1, 2, 3				H1, H2, H3
		2nd symmetric	alternate	H=H, 1=1	H1H1s, H2H1s, H2H2s, H3H2s
		2nd asymmetric	similar	HxH, 1x1	H1H1a, H2H1a, H2H2a, H3H2a
	**Secure polyfilament half-knot sequences: H2H1sH1s, H2H2s or H2H2a, H3H2s or H3H2a**				
**Half-hitch**	1, 2				S(1), (S2)
		2nd symmetric	alternate		
		sliding		S=S, 1=1	SSs(s)
		blocking		S//S	SSsb
		2nd asymmetric	similar		
		sliding		SxS, 1x1	SSa(s)
		blocking		S//xS	SSab
	**Secure polyfilament half-hitch sequences: SSsSabSab**				

## Data Availability

Original data will be made available by simple request to the corresponding author.
